# The MOBIOS^+^: A FAIR (Findable, Accessible, Interoperable and Reusable) database for Mindanao's terrestrial biodiversity

**DOI:** 10.3897/BDJ.11.e110016

**Published:** 2023-10-09

**Authors:** Krizler Cejuela Tanalgo, Kier Celestial Dela Cruz, Angelo Rellama Agduma, Jeaneth Magelen V. Respicio, Sumaira S. Abdullah, Renee Jane Alvaro-Ele, Bona Abigail Hilario-Husain, Meriam Manampan-Rubio, Sedra A. Murray, Lothy F. Casim, Athea Mohidda M. Pantog, Shiela Mae P. Balase, Rallyessa Mohann A. Abdulkasan, Chasty Andrea S. Aguirre, Nadjmussahar L. Banto, Sheila Mae M. Broncate, Ace D. Dimacaling, Gerald Vince N. Fabrero, Asraf K. Lidasan, Analiza A. Lingcob, Ariane M. Millondaga, Kathlene Faye L. Panilla, Crystal Queen M. Sinadjan, Norlaine D. Unte

**Affiliations:** 1 Ecology and Conservation Research Laboratory (Eco/Con Lab), Department of Biological Sciences, College of Science and Mathematics, University of Southern Mindanao, Kabacan, Philippines Ecology and Conservation Research Laboratory (Eco/Con Lab), Department of Biological Sciences, College of Science and Mathematics, University of Southern Mindanao Kabacan Philippines; 2 Molecular Parasitology Research Laboratory, Department of Biological Sciences, College of Science and Mathematics, University of Southern Mindanao, Kabacan, Philippines Molecular Parasitology Research Laboratory, Department of Biological Sciences, College of Science and Mathematics, University of Southern Mindanao Kabacan Philippines

**Keywords:** biodiversity records, conservation, FAIR database, islands, Philippines, shortfalls

## Abstract

**Background:**

Due to its complex geological history and the emergence of various biogeographic regions, the Philippines boasts an extraordinary array of flora and fauna. This unique combination has contributed to the country’s exceptional density of terrestrial species, making it amongst the highest in the world. Mindanao, in the southern part of the Philippines, is the second largest group of islands and supports high levels of endemism and proportion of threatened species. However, consolidated biodiversity records and information remain unavailable for the region. The primary goal of the Mindanao Open Biodiversity Information (MOBIOS^+^) database is to address these substantial data deficiencies by compiling contemporary biodiversity information from the 21^st^ century. This initiative seeks to enhance our comprehension of biodiversity trends in Mindanao over temporal and spatial dimensions, while also creating an openly-accessible database. The database we present here is the first of its kind and currently the most comprehensive attempt to establish the largest consolidated database for Mindanao biodiversity, based on publicly available literature. With its vast collection of biodiversity data, this database will prove to be a valuable resource for advancing biodiversity research and analysis. It will further facilitate the identification of species and areas that require immediate conservation prioritisation and action, addressing the urgent challenges posed by our rapidly changing planet.

**New information:**

The MOBIOS^+^ database is the first attempt to create a massive FAIR database aiming to collate biodiversity records from published literature in the Mindanao faunal region, south of the Philippines. The database currently includes 12,813 georeferenced specimen occurrences representing 1,907 unique taxa across 10 animal classes inhabiting the terrestrial and freshwater environments of Mindanao faunal region. We made all georeferenced specimen occurrences available in the Global Biodiversity Information Facility (GBIF) platform.

## Introduction

The current global biodiversity is at risk and faces an extinction rate higher than that of previous extinction events (Cowie et al. 2017). To tackle the pressing issue of biodiversity crisis, conservation biologists and experts must establish priorities to identify species and areas that require immediate attention and conservation efforts (Brum et al. 2017). Setting fine-scale prioritisation analysis is essential to ensure it complements the local biodiversity context and reflects global priorities (Knight et al. 2008, Tanalgo and Hughes 2019). Effective biodiversity synthesis and prioritisation on any scale require good data integration to accurately identify priorities, based on data and evidence (Heberling et al. 2021, Dela Cruz et al. 2023).

The Philippine Archipelago's more than 7,100 islands, has one of the highest levels of endemism of any place and is a hotspot for biodiversity conservation, directly related to the tropical environment of the country and the complicated geological past (Rickart et al. 2003, Lohman et al. 2010). The three largest faunal regions in the Philippines are the Luzon faunal region, the Mindanao faunal region and the Palawan faunal region (Heaney 1986). This study mainly focused on the Mindanao faunal region, encompassing the Visayas, specifically the Eastern Visayas, which includes the Provinces of Samar, Leyte, Bohol and smaller islands such as Basilan, Dinagat and Surigao. Although Mindanao is the second largest group of islands in the Philippines next to Luzon, biodiversity studies in the region have been limited in recent decades and are often constrained by low research funding allocation, a low number of experts and the peace and order situation that hinder researchers in exploring many potential areas for biodiversity studies (Amoroso 2000, Alcala 2004, Agduma et al. 2023). With the advent of new technological advances, increasing collaborations and the emergence of diverse and young biodiversity researchers, the number of biodiversity research has increased in the early 21^st^ century (Abdullah et al. 2023, Agduma et al. 2023, Dela Cruz et al. 2023). Integrating this unconsolidated biodiversity information is another new challenge that has a crucial impact on understanding the large-scale biodiversity patterns in Mindanao. These gaps challenge the realisation of practical, data-driven and up-to-date conservation priorities, especially for the region’s endemic and threatened species.

**Figure 1.**
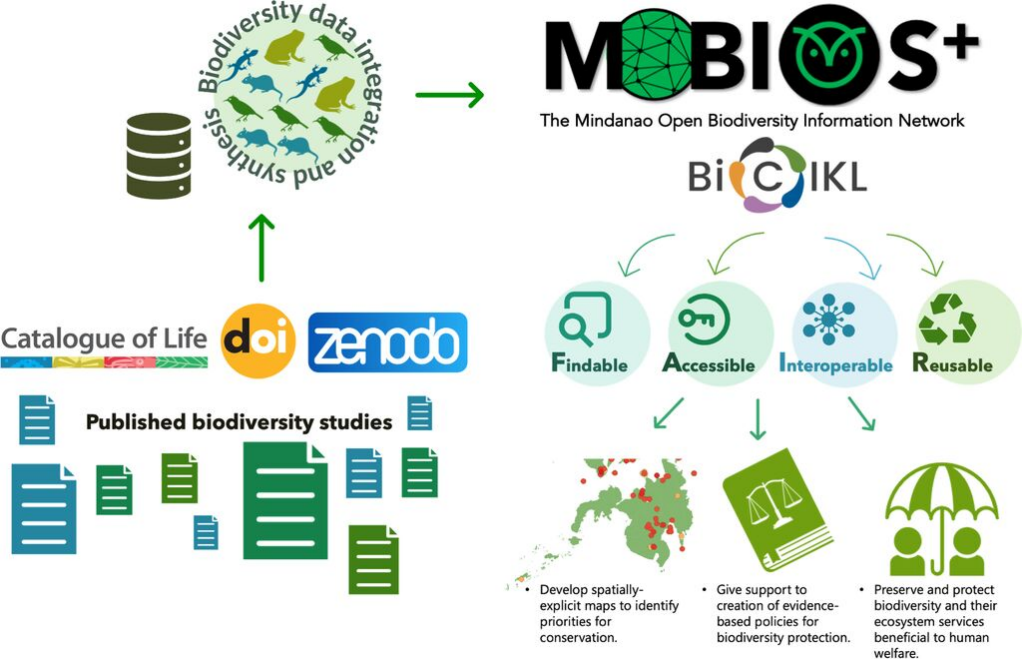
A proposed framework for academic and research institutions to address and advance biodiversity knowledge gaps and strengthen the availability and access to biodiversity information that will facilitate effective conservation prioritisation through FAIR data sharing.

In this data paper, we introduce the Mindanao Open Biodiversity Information (MOBIOS^+^) database, which aims to create a FAIR (Findable, Accessible, Interoperable and Reusable) platform by integrating publicly-accessible biodiversity data from published studies within the Mindanao faunal region (Fig. 1). By leveraging the integrated data within MOBIOS^+^, researchers can gain a comprehensive understanding of biodiversity distribution, patterns and threats within Mindanao and the wider Philippines across different scales. The valuable information provided by the database will prove instrumental in the development of biodiversity synthesis, modelling, mapping and prediction of environmental changes specifically in the Mindanao Region.

## General description

### Purpose

The MOBIOS^+^ project has an overarching goal of establishing a biodiversity database for Mindanao following the FAIR (Findable, Accessible, Interoperable and Reusable) principles to advance studies in biodiversity and develop a synthesis to identify current and future conservation priorities in the region. This involves consolidating species occurrence records from studies conducted in different institutions across Mindanao into an open and readily-accessible platform (Fig. 1). To do this, we collated biodiversity records from published studies and organised them in a standard format, machine-readable and digitally available through the Global Biodiversity Information Facility (GBIF) platform. This project aims to continuously update the species database, complementing on-ground biodiversity efforts in Mindanao. The database is housed at the Biodiversity Synthesis Centre of the Ecology and Conservation (Eco/Con) Research Laboratory at the Department of Biological Sciences, University of Southern Mindanao.

## Sampling methods

### Study extent

https://scholar.google.com.ph/The MOBIOS^+^ database currently contains information for 12,813 georeferenced specimen occurrences from 1,907 species within the Mindanao Faunal region (Mainland Mindanao and adjacent provinces in Visayas). The database represents at least ten taxonomic classes of terrestrial and freshwater fauna. This is the first database version that contains biodiversity records based on literature from the early 21^st^ century for terrestrial fauna from the faunal region of Mindanao.

### Sampling description

We applied the PRISMA (Preferred reporting items for systematic reviews and meta-analyses) approach (O'Dea et al. 2021) to collect and screen biodiversity studies from Mindanao from 2000-2023. We used the combinations of the following keywords: ‘Biodiversity stud*’, ‘Assessment*, ‘Survey’, ‘Terrestrial’, ‘Freshwater’ and ‘Mindanao’ to search for published literature from Google Scholar (https://scholar.google.com.ph/). We also explored the Biodiversity Literature Repository (BLR) (https://biolitrepo.org/) and the self-archiving ResearchGate (https://www.researchgate.net/) for additional published studies, particularly on new localities and natural history notes of species. We searched for literature related to surveys of terrestrial vertebrates (e.g. amphibians, reptiles, birds and mammals), fishes, gastropods, crustaceans, bivalves, insects and arachnids (see examples in: Abdullah et al. (2023), Dela Cruz et al. (2023)). We then assigned teams to collate and analyse data for specific taxonomic groups. We sampled each literature paper for the location of species occurrence (lattitude and longitude) from published articles and books using the Darwin Core Biodiversity Standard format (Wieczorek et al. 2012). We extracted information for the species list, taxonomic classification, conservation status, location of the species and other relevant information.

### Quality control

We counter-checked and curated all listed species and their distribution in the database using the Integrated Taxonomic Information System (ITIS) database (https://www.itis.gov/). We excluded dubious species, such as those with problematic identification or species that naturally do not occur in the range without proper discussions or expert clarifications. Species taxonomic names were aligned and standardised following the updated version Catalogue of Life (CoL) (https://www.catalogueoflife.org). We retained the taxonomic classification of species with confusing arrangements. We plotted and mapped all species occurrence within the boundaries of the Mindanao faunal region using QGIS (v. 3.30) (QGIS team 2023) to curate species occurrence within the geographical range. Biodiversity records outside the range or within unusual locations were counter-checked with the original reference and corrected. We will update the MOBIOS^+^ database on an annual basis following the same screening procedure and standards. A release note will be published by the corresponding author and institution when the new version of the database is released.

### Step description

The data included in the MOBIOS^+^ database were extracted from published research articles publicly available online and organised using the GBIF standards for data publication. The following steps were taken to audit the accuracy of the dataset in the MOBIOS^+^ database: (1) Collating and filtering published biodiversity studies from the Mindanao faunal region; (2) Reviewing the studies for suitability based on criteria; (3) Extracting species occurrence data and other relevant metadata from biodiversity studies in the Mindanao faunal region; (4) Placing the species distribution and other metadata in Microsoft Office Excel format; (5) Curating species occurrences in Quantum GIS; (6) Organising of the occurrence dataset following Darwin Core Standards; (7) Literature without permanent identifiers was provided with DOIs by uploading a copy of the literature in the Biodiversity Library Repository within Zenodo.

## Geographic coverage

### Description

The database contains data for terrestrial and freshwater ecosystems in the entire faunal region of Mindanao, south of the Philippines.

### Coordinates

4.7438 and 12.4646 Latitude; 126.6303 and 118.4747 Longitude.

## Taxonomic coverage

### Description

**Figure 2.**
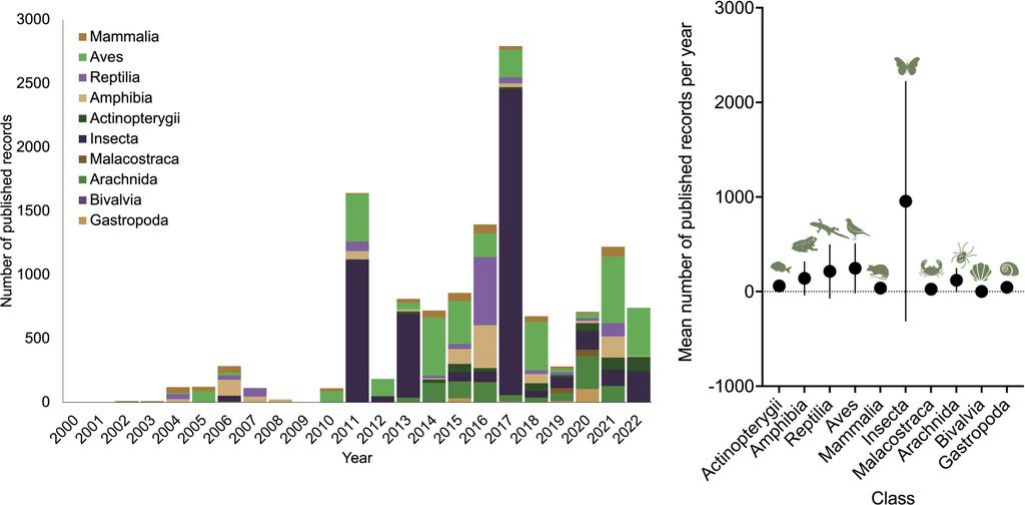
Distribution of biodiversity records across years and taxonomic groups from published papers.

**Figure 3.**
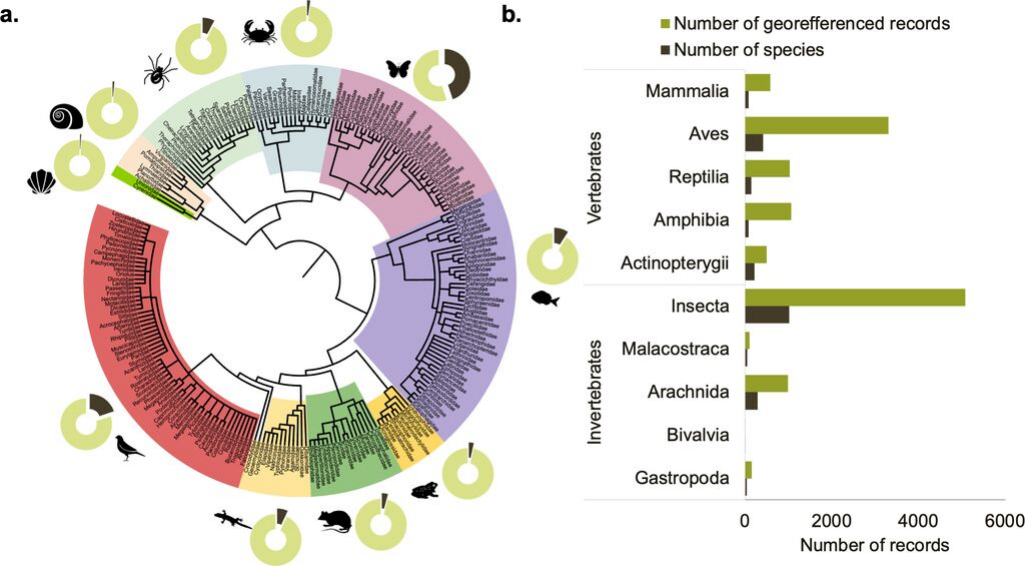
Diversity and distribution of species occurrence records across taxonomic groups included in the first version of the MOBIOS^+^ database. The diversity of species (percentage, %) according to class compared to the overall number of species recorded in the MOBIOS^+^ database (a); and the total number of species and the number of georeferenced occurrences per animal class (b).

**Figure 4.**
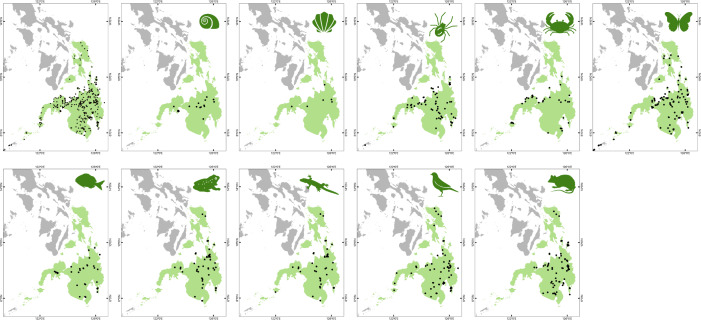
Visualised georeferenced species occurrences in the Mindanao faunal region currently included in the MOBIOS^+^ database (version 1).

The 12,813 georeferenced species occurrences from published papers comprise 1,907 species from 10 taxonomic classes (Figs 2, 3). Invertebrates are represented by Gastropoda (27 spp., 1%), Bivalvia (4 spp., 0.20%), Arachnida (149 spp., 8%), Malacostraca (43 spp., 2%) and Insecta (866 spp., 45%) (Fig. 4), whilst the vertebrates are represented by Actinopterygii (176 spp., 9%), Amphibia (65 spp., 3%), Reptilia (135 spp., 7%), Aves (382 spp., 20%) and Mammalia (78 spp., 4%).

### Taxa included

**Table:** 

Rank	Scientific Name	Common Name
kingdom	Animalia	Animals
class	Gastropoda	Gastropods
class	Bivalvia	Bivalves
class	Arachnida	Arachnids
class	Malacostraca	Crustaceans
class	Insecta	Insects
class	Actinopterygii	Fishes
class	Amphibia	Amphibians
class	Reptilia	Reptiles
class	Aves	Birds
class	Mammalia	Mammals

## Temporal coverage

**Data range:** 2000-1-01 – 2022-12-31.

## Usage licence

### Usage licence

Open Data Commons Attribution License

## Data resources

### Data package title

MOBIOS^+^ Data set version 1

### Resource link

https://ipt.pensoft.net/resource?r=mobios_data

### Number of data sets

1

### Data set 1.

#### Data set name

The MOBIOS^+^ database: a FAIR Mindanao Biodiversity

#### Data format

Darwin Core Archive format

#### Description

Our dataset contains 12,813 occurrence data points for 1,907 taxonomic species from 10 classes recorded within Mindanao fauna region of the Philippines.

**Data set 1.** 

Column label	Column description
occurrenceID	An identifier for the Occurrence (as opposed to a particular digital record of the occurrence). In the absence of a persistent global unique identifier, construct one from a combination of identifiers in the record that will most closely make the occurrenceID globally unique.
basisOfRecord	The specific nature of the data record.
occurrenceStatus	A statement about the presence or absence of a Taxon at a Location.
year	The four-digit year in which the Event occurred, according to the Common Era Calendar.
continent	The name of the continent in which the Location occurs.
countryCode	The standard code for the country in which the Location occurs.
stateProvince	The name of the next smaller administrative region than country (state, province, canton, department, region etc.) in which the Location occurs.
county	The full, unabbreviated name of the next smaller administrative region than stateProvince (county, shire, department etc.) in which the Location occurs.
municipality	The full, unabbreviated name of the next smaller administrative region than county (city, municipality etc.) in which the Location occurs.
locality	The specific description of the place where the species was recorded.
decimalLatitude	The geographic latitude (in decimal degrees, using the spatial reference system given in geodeticDatum) of the geographic centre of a Location.
decimalLongitude	The geographic longitude (in decimal degrees, using the spatial reference system given in geodeticDatum) of the geographic centre of a Location.
coordinateUncertaintyInMetres	The horizontal distance (in metres) from the given decimalLatitude and decimalLongitude describing the smallest circle containing the whole of the Location.
geodeticDatum	The ellipsoid, geodetic datum or spatial reference system (SRS) upon which the geographic coordinates given in decimalLatitude and decimalLongitude are based.
georeferencedBy	A list (concatenated and separated) of names of people, groups or organisations who determined the georeference (spatial representation) for the Location.
scientificName	The full scientific name in lowest level taxonomic rank that can be determined.
kingdom	The full scientific name of the kingdom in which the taxon is classified.
phylum	The full scientific name of the phylum or division in which the taxon is classified.
class	The full scientific name of the class in which the taxon is classified.
family	The full scientific name of the family in which the taxon is classified.
genus	The full scientific name of the genus in which the taxon is classified.
specificEpithet	The name of the first or species epithet of the scientificName.
taxonRank	The taxonomic rank of the most specific name in the scientificName.
scientificNameAuthorship	The authorship information for the scientificName formatted according to the conventions of the applicable nomenclaturalCode.
taxonomicStatus	The status of the use of the dwc:scientificName as a label for a taxon.
associatedReferences	A list (concatenated and separated) of identifiers (publication, bibliographic reference, global unique identifier, URI) of literature associated with the Occurrence.

## Additional information

The MOBIOS^+^ primary objective is to make biodiversity information from the literature more accessible and ready and ensure the reusability of the data within our database for various biodiversity research purposes. To achieve this, we linked the MOBIOS^+^ database deposited in GBIF IPT to the Catalogue of Life (CoL) (https://www.catalogueoflife.org/) and the original sources of the species records using the Digital Object Identifier (DOI) associated with each reference (see Fig. 1) (Table 1). We assigned permanent object identifiers (DOIs) to those items that initially lacked them, utilising the Biodiversity Library Repository within Zenodo. The infrastructure utilises the latest version of the unique identifier which the updated Catalogue of Life (CoL) provides to establish links between species (Suppl. material 1).

### Important caveats for Mindanao's biodiversity database

Based on our current work, we acknowledge some crucial gaps and caveats that limit the use of our database, but will be addressed in the future development and progress of the MOBIOS+ database.

1. Our current database contains limited set of occurrence data from faunal groups. Future developments and progress of the MOBIOS^+^ will focus on filling this gaps by including other taxonomic groups (e.g. invertebrates, plants and fungi) in the database.
2. Mindanao has been a significant hub for biodiversity research, with many species recorded in the past two-decades. However, we also observed that many studies are limited to inventories or rapid assessments (e.g. Tanalgo and Hughes 2018). Although the results of these studies are useful, there is a lack of focus on the ecological aspects of many species and their habitats, which are equally important in developing effective species conservation approaches. This may be a common issue for many taxonomic groups in Mindanao and throughout the country.
3. Although there is a significant amount of good data generated from various field studies, they were often poorly analysed and discussed (Tanalgo et al. 2019, Gracia Jr. et al. 2021). Many of these datasets could provide more information and improve understanding, but they remain under-utilised and are simply published.
4. Most fieldwork studies were not standardised, for example, sampling designs and methods, making comparative spatiotemporal monitoring difficult. We suggest and encourage biodiversity scientists to implement more standardised, transparent and reproducible field methods in future biodiversity surveys.
5. Many useful biodiversity data are present in published literature, but numerous articles have been published in less visible journals (e.g. predatory journals) and most without permanent identifiers (e.g. no DOIs). We propose that biodiversity researchers should be more selective in their choice of journals when publishing their biodiversity data and inventories (i.e. use of journals that promotes FAIR and open-biodiversity data sharing).
6. There were good capacity building programmes amongst scientists and young generations in the recent years in Mindanao. Yet, there are only a few collaborative biodiversity studies amongst academic institutions in Mindanao. This is observed in a few papers with co-authorship from multiple institutions and a small group of academics dominates authorship. We suggest implementing more collaborative and open network of biodiversity expertise and data-sharing amongst academics and practioners within and outside Mindanao (see Agduma et al. (2023)).

Mindanao is rich in biodiversity data, but significant challenges exist in analysing and using these data. To address these challenges and shortfalls, there is a need for implementation of better standardisation and collaboration between academic institutions and for the development of more FAIR biodiversity databases.

## Supplementary Material

9D0E1DFE-F35F-537F-A7C2-0924869DCE3E 10.3897/BDJ.11.e110016.suppl1 Supplementary material 1 Supplementary file for occurrence and species records from MOBIOS^+^ database Data type occurrences Brief description This supplementary file contains linked data from Catalogue of Life (CoL) and the DOI of the associatedReference. File: oo_915302.xlsx https://binary.pensoft.net/file/915302 Krizler C. Tanalgo and MOBIOS consortium

## Figures and Tables

**Table 1.** Additional information for linked data to unique identifiers

Column	Definition
CoLIdentifiers	Catalogue of Life (CoL) identifiers; the Stable name identifiers for the Catalogue of Life (CoL).
CoLLinked	Linked to the CoL record.
DigitalObjectIdentifier	The permanent object identifier of the associatedReferences.
